# A screening and brief intervention for women in OB/GYN care

**DOI:** 10.1186/1940-0640-10-S2-O4

**Published:** 2015-09-24

**Authors:** Tatiana Balachova, Mark Chaffin, Barbara Bonner, Galina Isurina, Larissa Tsvetkova, Elena Volkova

**Affiliations:** 1 Pediatrics, The University of Oklahoma Health Sciences Center, Oklahoma City, USA; 2 School of Public Health, Georgia State University, Atlanta, USA; 3 St. Petersburg State University, St. Petersburg, Russia; 4 Nizhny Novgorod State Pedagogical University, Nizhny Novgorod, Russia

## Background

Alcohol consumption levels in Russia are among the highest in the world[1]. Fetal Alcohol Spectrum Disorders in children are completely preventable by avoiding alcohol use during pregnancy[2]; yet, substantial numbers of women around the world consume alcohol during pregnancy mostly prior to pregnancy recognition[3-5]. A U.S. prevention model, Project CHOICES, utilized a pre-conceptional approach consisting of four counseling sessions and a family planning clinic visit[6]. A brief intervention protocol[7] and the CHOICES were adapted by the research team to design a brief intervention for implementation in public OB/GYN clinics in two regions in Russia. The objective of this study was to evaluate impact of the adapted protocol in reducing the risk for alcohol-exposed pregnancies (AEP) and alcohol consumption in general.

## Material and methods

A dual-focused (alcohol use and the risk for an unplanned pregnancy) brief physician intervention (DFBPI) was designed to be delivered by OB/GYN physicians[8]. The intervention consisted of two face-to-face, 5-minute sessions incorporated into routine clinic visits. A two-arm cluster-randomized controlled trial of the DFBPI was conducted at 20 district OB/GYN clinics. A total of 767 non-pregnant women, aged 18-44 years, at risk for an AEP (at-risk drinking, heterosexual intercourse, and the inconsistent use of contraception) were recruited for the study. Data included 90-day retrospective Time-Line Follow Back (TLFB) reports of daily alcohol consumption at baseline, 3, 6, and 12-months. Drinking trajectories were modeled using three-level semi-continuous piecewise latent trajectory models, with post-intervention intercepts and slopes conditional on treatment assignment. A subpopulation analysis was conducted for women who became pregnant during the 12 month interval for alcohol consumption prior to and after pregnancy recognition. Latent class transition models from baseline to post-intervention were tested to identify shifts in drinking patterns conditional on intervention condition.

## Results

Physicians were able to implement, maintain skills, and deliver DFBPI. During the course of the study, 72 participants became pregnant. Participants in the DFBPI condition showed a larger decrease in alcohol consumption immediately following the intervention, which was maintained over the follow-up period. Newly pregnant women in the intervention condition showed a substantial drop in alcohol consumption in the weeks prior to pregnancy recognition, consistent with the targeted purpose of the intervention. Women in the intervention condition were more likely to transition out of a high alcohol use pattern and into a lower use pattern rather than into a binging pattern.

## Conclusions

Results support the feasibility and efficacy of the DFBPI at OB/GYN clinics.

## Trial Registration

NCT01961050
